# Autologous CIK cells combined with chemotherapy as the first-line treatment for locally advanced or metastatic gastric cancer is safe and feasible

**DOI:** 10.3389/fimmu.2023.1267369

**Published:** 2023-11-01

**Authors:** Xiaoting Ma, Liming Peng, Junqing Wang, Lizhen Gao, Wen Zhang, Xu Lu, Jingwei Liu, Lin Yang

**Affiliations:** ^1^ Department of Medical Oncology, National Cancer Center/National Clinical Research Center for Cancer/Cancer Hospital, Chinese Academy of Medical Sciences and Peking Union Medical College, Beijing, China; ^2^ Department of Medical Oncology, Beijing Chaoyang Huanxing Cancer Hospital, Beijing, China; ^3^ Department of Oncology, Beijing Biohealthcare Biotechnology Co., Ltd, Beijing, China

**Keywords:** adverse event, CIK, gastric cancer, overall survival, progression-free survival

## Abstract

**Aim:**

To evaluate the safety and initial efficacy of autologous cytokine-induced killer (CIK) cells combined with S-1+oxaliplatin (SOX) as the first-line treatment for locally advanced or metastatic gastric cancer (GC).

**Materials and methods:**

In this two-arm, single-center exploratory trial, patients with locally advanced or metastatic GC were randomly assigned (1:1) to receive autologous CIK cells in combination with SOX (CIK-SOX) or SOX alone. The primary endpoint was the incidence of adverse events (AEs). Progression-free survival (PFS), overall survival (OS), objective response rate (ORR), and disease control rate (DCR) served as the secondary endpoints.

**Results:**

Fifty-nine patients were enrolled in the study between November 20, 2014 and September 6, 2017. A total of 31 patients received CIK-SOX and 28 patients received SOX. The most common AEs in both groups were gastrointestinal reaction, leucopenia, neutropenia, anemia, thrombocytopenia, hyperbilirubinemia, and elevated aspartate transaminase concentration, with a higher incidence of these conditions in the SOX group. The median PFS for the CIK-SOX and SOX groups was 6.9 and 4.9 months, respectively (hazard ratio (HR) 0.80, *p*=0.45). The respective median OS values were 17.8 and 9.75 months (HR 0.76, *p*=0.34). Patients who received more than three injections of specific lymphocyte subsets benefited the most from this combination therapy. Cox univariate and multivariate analyses showed that tumor metastasis to more than two organs was the main risk factor for PFS and OS. A total of 29 patients in the CIK-SOX group and 25 in the SOX group had measurable lesions. The ORR for the CIK-SOX and SOX groups was 55.2% and 32.0%, while the DCR was 93.1% and 88.0%, respectively.

**Conclusion:**

The safety of CIK-SOX as the first-line treatment for patients with locally advanced or metastatic GC was good. Although the PFS and OS in the CIK-SOX group were not statistically significantly different compared to the values in the SOX alone group, this treatment increased the PFS and OS duration, with the absolute improvement in OS of about 8.05 months. Continuous benefit from the CIK-SOX treatment was observed during long-term follow-up.

**Clinical trial registration:**

https://clinicaltrials.gov/study/NCT02504229?term=NCT02504229&rank=1, identifier ChiCTR-IPR-15005923; NCT02504229.

## Introduction

Gastric cancer (GC) is one of the most common malignant tumors. Its incidence ranks fifth in the world, while death rate ranks second, representing a serious threat to people’s life and health (1). Surgical resection is the main radical treatment method for GC, although less than 50% of patients can achieve R0 resection (2). More than 80% of GC patients are in the advanced stage of the disease at the time of its discovery. For patients with advanced locally advanced or metastatic GC, platinum combined with fluorouracil is the main first-line treatment. S-1 is a fluorouracil derivative oral anticancer agent that has shown promising efficacy in GC populations in Asia. However, with the increasing resistance of GC cells to chemotherapy drugs, it is often difficult for patients with locally advanced or metastatic GC to benefit from chemotherapy (3–5). Although the use of immune checkpoint inhibitors (ICIs) has survival benefits in patients with locally advanced or metastatic GC, their efficacy is not satisfactory and new treatment methods are urgently needed to improve the prognosis of patients with locally advanced or metastatic GC (6–8).

Adoptive cell therapy (ACT) aims to collect human autoimmune cells, expand their number after cultivation *in vitro*, increase their targeted killing function, and then return them to the patient’s body to kill pathogens, cancer cells, or mutated cells in blood and tissues. The current ACT includes tumor-infiltrating lymphocytes, lymphokine-activated killer cells, dendritic cells (DCs), natural killer (NK) cells, and cytokine-induced killer (CIK) cells, as well as chimeric antigen receptor (CAR) T cells and T cell receptor (TCR) engineered T cells. These cells are predominantly lymphocytes and play a vital role in tumor microenvironment. In addition, many studies have confirmed that dendritic cell - cytokine-induced killer (DC-CIK) cells, chimeric antigen receptor - natural killer (CAR-NK) cells, and other modes of ACT can improve the proliferation rate and killing activity of cells and make the killing effect on tumor cells more specific. In addition, this treatment can kill and remove extremely small tumor foci that cannot be resected surgically or scattered tumor cells in the body and play a role in delaying or preventing tumor metastasis or recurrence (9–11). They can be combined with other treatments to improve survival rates in patients with cancer. Studies have shown that ACT has been applied in many solid tumors, including melanoma, lung cancer, and cervical cancer, and sustained tumor regression has been observed (12–15). Therefore, the potential of ACT in combination with traditional therapies to reduce recurrence and metastasis of malignant tumors deserves further exploration.

At present, a variety of ACTs combined with chemotherapy have been confirmed to be effective and safe in the treatment of locally advanced or metastatic GC, but no consensus has been reached (16–18). The present study compared autologous CIK cells combined with S-1 + oxaliplatin (SOX) and SOX alone to explore the safety and effectiveness of autologous CIK cells combined with SOX (CIK-SOX) as the first-line treatment for locally advanced or metastatic GC patients.

## Patients and methods

### Study design

This randomized, open-label, exploratory study was conducted at a single center. Since the main purpose of the study was exploratory, no statistical assumptions were made about the sample size. Patients were randomly assigned (1:1) to receive CIK-SOX or SOX alone using the central randomization system. Randomized grouping was stratified according to the Eastern Cancer Cooperation Group (ECOG) performance status (PS) score of 0 or 1 and the current disease stage (locally advanced or metastasis). Six cycles of the SOX treatment were administered. Patients without progressive disease (PD) received autologous CIK cells combined with S-1 or S-1 as the maintenance treatment until PD or unacceptable toxicity level was achieved, or out of the group. No crossing between the two groups is allowed.

The present study (ChiCTR-IPR-15005923; NCT02504229, https://clinicaltrials.gov/study/NCT02504229?term=NCT02504229&rank=1) was approved by the Ethics Committee of the National Cancer Center/Cancer Hospital, the Chinese Academy of Medical Sciences and Peking Union Medical College and was conducted in accordance with the principles of Declaration of Helsinki and the International Conference on Harmonization Good Clinical Practice guidelines. All patients gave written informed consent before enrollment.

### Patients

Patients with histologically confirmed gastric adenocarcinoma and radiographic diagnosis of locally advanced or metastatic GC were eligible for inclusion. Other inclusion criteria included age of ≥ 18 years old, measurable and/or evaluable lesions according to the Response Evaluation Criteria in Solid Tumors (RECIST) guideline version 1.1, ECOG PS score of < 2, ability to take medications orally, no previous palliative chemotherapy (adjuvant chemotherapy or neoadjuvant chemotherapy with interval of ≥ 6 months was allowed), time since last radiotherapy treatment of ≥ 3 weeks, expected survival period of ≥ 3 months, and no severe lung, heart, or any other comorbidities. The following conditions for blood test examinations had to be satisfied within 14 days before enrollment to verify proper organ function: white blood cell count of (3.0–10.0) × 10^9^/L, lymphocyte count of ≥ 0.8 × 10^9^/L, neutrophil count of ≥ 1.5 × 10^9^/L, platelet count of ≥ 100 × 10^9^/L, serum aspartate aminotransferase and alanine aminotransferase level < 2.5 times of the normal limit, serum total bilirubin level < 1.5 times of the normal limit, serum creatinine level < the upper limit of normal limit, and creatinine clearance of ≥ 50 mL/min. The main exclusion criteria included uncontrolled medical conditions, pleural effusion or ascites to degree which require drainage within 2 weeks of enrollment, no other malignant tumors in the last five years, continuous systemic steroid administration, peripheral neuropathy of grade 2 or above, pregnancy or lactation, or failure to follow up regularly as planned for any reason.

### Preparation of autologous CIK cells

CIK cells were derived from the patient’s own peripheral blood. Peripheral blood mononuclear cells were isolated using Ficoll-Hypaque density gradient centrifugation. Mononuclear cells (lymphocytes) were adjusted to a concentration of 2×10^6^ cells/mL and cultured in complete medium (Lonza, Basel, Switzerland). Next, 5% heat-inactivated autologous plasma (Bio-Techne, Minnesota, America) containing human interferon gamma (IFN-γ) was added. On the first day, the medium was supplemented with 100 ng/mL of anti-CD3 monoclonal antibody (Miltenyi Biotec, Bergisch-Gladbach, Germany) and 500 U/mL of IL-2 (SL Pharmaceutical Co., Ltd, Beijing, China). Cell density was adjusted to 1×10^6^/mL on days 4, 7, and 11 and the cells were re-stimulated with 50 ng/mL of IL-15 (Bio-Techne) (19–21). After the addition of anti-CD3 monoclonal antibody, the lymphocytes were collected after 14 days of culture and tested for bacteria, mycoplasma, and endotoxins to ensure that the lymphocytes were pollutant-free.

### Cell infusion

After 14 days of cultivation, the proportion of obtained CD4, CD8, and NKT cells was as follows: CD3+: 85–98.3%, with an average of 92.6%; CD3+CD8+: 49.6–81.8%, with an average of 68.2%; CD3+CD4+: 9.7–43.5%, with an average of 22.7%; CD3-CD56+: 1–15%, with an average of 6.0%; and CD3+CD56+(NKT): 3.4–27.4%, with an average of 13.4%. The final cell products in all patients were highly viable (95%) and uncontaminated. After completing quality testing, all qualified immune cells were infused back into the patients. All patients in the CIK group completed at least one cycle of immune cell infusion containing an average of 8.6×10^9^ cells per treatment.

### Therapeutic regimen

Patients received SOX alone or in combination with autologous CIK cells every 3 weeks. SOX regimen included oxaliplatin (130 mg/m^2^; 2-h intravenous infusion on day 1) and S-1 taken orally at doses of 80, 100, or 120 mg every day depending on body surface area (< 1.25 m^2^, 1.25–1.5 m^2^, or > 1.5 m^2^) on days 1 to 12. The combination therapy group was administered autologous CIK cell therapy on day 14. Autologous CIK cell therapy started on the first cycle. SOX regimen lasted for six cycles. Patients without PD received S-1 combined with autologous CIK cells (combination therapy group) or S-1 monotherapy (chemotherapy group) as maintenance therapy until achieving PD or intolerable toxicity, withdrawal of informed consent, or death. Second-line treatment was not predetermined. The researchers followed agency guidelines for antiemetic premedication and growth factor use. The chemotherapy dose was reduced at the discretion of the investigator.

### Assessment

Clinical examination and laboratory evaluation were required before each treatment cycle. After baseline assessment, tumor status was assessed using computed tomography scanning and tumor markers every 6 weeks until achieving PD according to the RECIST guideline version 1.1 (https://www.cancer.gov/).

Safety was evaluated based on adverse event (AE) reports, laboratory test results, and vital sign measurements. AE evaluation was classified according to the common terminology criteria for adverse events (CTCAE) version 4.0, where level 1 indicates a mild AE, level 2 indicates a moderate AE, level 3 indicates a serious AE or one that is medically significant but not immediately life-threatening, level 4 indicates a life-threatening AE, and level 5 indicates death.

### Statistical analysis

The purpose of the present study was to explore the safety and effectiveness of the CIK-SOX regimen. The primary end point was the incidence of AEs. The AE evaluation was calculated and classified according to CTCAE v4.0. The secondary end points were progression-free survival (PFS), overall survival (OS), objective response rate (ORR), and disease control rate (DCR). PFS was defined as the earliest evidence from randomization to PD (according to RECIST v1.1) or the time of death from any cause, whichever occurred first. OS was defined as the time from randomization to death for any reason. Patients who survived and did not progress at the last disease assessment were reviewed. ORR was defined as the proportion of patients with complete remission (CR) or partial remission (PR). DCR was defined as the proportion of patients with CR, PR, or stable disease (SD). The safety endpoint was based on the safety set (SS), in which the patient received at least one protocol treatment and one safety evaluation. The efficacy end point was based on the full analysis set (FAS), in which the population received at least one protocol treatment and had one tumor evaluation. The median follow-up period of the entire study cohort was calculated according to the reverse Kaplan-Meier method. Kaplan-Meier survival curve was used to estimate OS and PFS and log-rank test was used to evaluate the differences between treatment groups. The Cox proportional hazards model was used to estimate the risk ratio. All statistical tests were bidirectional, and *p* value of < 0.05 was considered to indicate statistical significance. R (version 4.0.5) and R studio were used for statistical analysis.

## Results

### Patient disposition and characteristics

**Figure 1:**
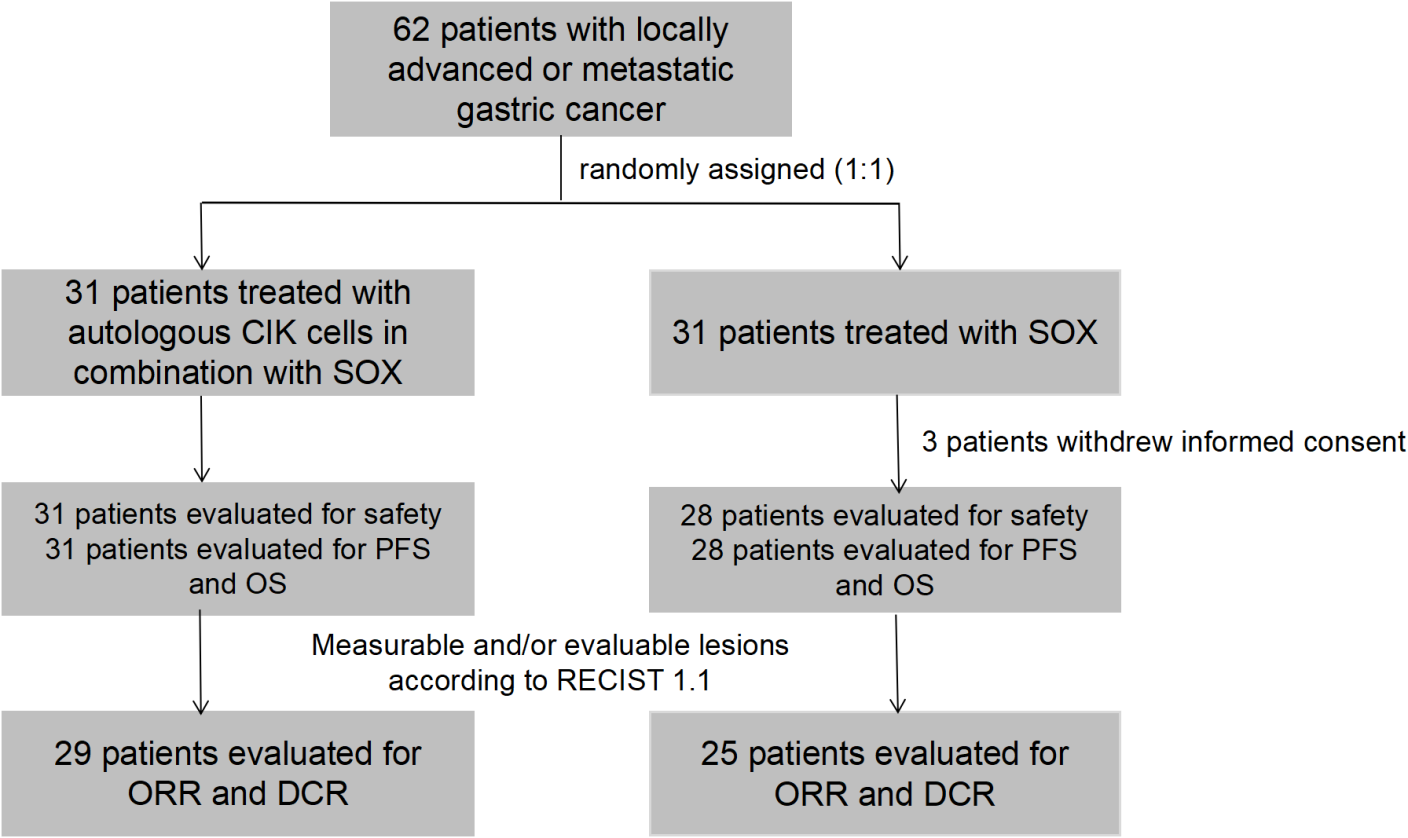
A flow diagram for the study. 62 patients were randomly assigned to receive autologous CIK cells in combination with SOX or SOX alone. 3 patients in SOX group withdrew their informed consent and did not start the study treatment. 31 patients in CIK-SOX group and 28 patients in SOX group were evaluated for safety and efficacy. 29 patients in the CIK-SOX group and 25 patients in the SOX group had measurable lesions, and assessed ORR and DCR. CIK, cytokine-induced killer; SOX, S-1 + oxaliplatin; CIK-SOX, autologous CIK cells in combination with SOX; RECIST, Response Evaluation Criteria in Solid Tumors; ORR, objective response rate; DCR, disease control rate.

Between November 20, 2014 and September 6, 2017, 62 patients were randomly assigned to receive treatment with CIK-SOX (n=31) or SOX alone (n=31). Three patients in the SOX group withdrew their informed consent and did not start the study treatment. Thus, SS and FAS included 31 patients in the CIK-SOX group and 28 patients in the SOX group (**Figure 1**). The demographic and baseline tumor characteristics of patients are presented in **Table 1**. More than 75% of patients were male, and more than 85% were <65 years old. Peritoneal metastasis occurred in 29% and 10.7% of patients in the CIK-SOX and SOX groups, respectively.

**Table 1:** Baseline characteristics.

Variable	All 59	CIK-SOX 31	SOX 28
Age	60(53,61)	58(54,60)	60(53,61)
Sex, n(%)			
Male	46(78.0)	25(80.6)	21(75.0)
Female	13(22.0)	6(19.4)	7(25.0)
ECOG-PS, n(%)			
0	17(28.9)	10 (32.3)	7 (25.0)
1	42(71.2)	21(67.7)	21(75.0)
TNM staging, n(%)			
III	2(3.4)	1(3.2)	1(3.6)
IV	57(96.7)	30(96.8)	27(96.4)
Disease status, n(%)			
Unresectable ^*^	51(86.4)	29(93.5)	22(78.6)
Recurrent ^#^	8(13.6)	2(6.5)	6(21.4)
Number of organs involved, n(%)			
0	2(3.4)	1(3.2)	1(3.6)
1	28(47.4)	12(38.7)	16(57.1)
≥2	29(49.2)	18(58.1)	11(39.3)
Site of metastasis, n(%)			
Lymph node	27(45.8)	12(38.7)	15(53.6)
liver	21(35.6)	12(38.7)	9(32.1)
lung	2(3.4)	1(3.2)	1(3.6)
Peritoneum	12(20.3)	9(29.0)	3(10.7)
Bone	6(10.2)	4(12.9)	2(7.1)
Other	9(15.3)	5(16.1)	4(14.3)

*The initial diagnosis was unresectable. ^#^ Radical gastrectomy was performed in the past, this time it was gastric cancer recurrence, and the recurrence location was distant organs.

SOX, S-1+oxaliplatin; CIK-SOX, CIK cells in combination with SOX; ECOG, Eastern Oncology Collaboration Group; PS, performance status.

### Treatment exposure

As of December 6, 2017, all 59 patients ceased study therapy, most of them due to PD. Furthermore, 61.3% (19/31) of patients in the CIK-SOX group and 53.5% (15/28) of patients in the SOX group received follow-up chemotherapy, while 12.9% (4/31) and 7.1% (2/28) of patients, respectively, received radical gastrectomy.

### Safety

**Table 2** shows the incidence of treatment-related AEs. The most common AEs in the two groups were gastrointestinal reaction, leucopenia, neutropenia, anemia, thrombocytopenia, hyperbilirubinemia, and elevated aspartate transaminase concentration. The incidence in the chemotherapy group was higher. Grade 3–4 AEs in the CIK-SOX group were neutropenia (four cases [12.9%]), anemia (one case [3.2%]), thrombocytopenia (three cases [9.7%]), febrile neutropenia (three cases [9.7%]), fever (one case [3.2%]), elevated total bilirubin (one case [3.2%]) and elevated direct bilirubin (one case [3.2%]). Grade 3–4 AEs in the SOX group were neutropenia (10 cases [35.7%]), febrile neutropenia (nine cases [32.1%]), anemia (two cases [7.1%]), thrombocytopenia (one case [3.6%]), elevated total bilirubin (one case [3.6%]) and elevated direct bilirubin (one case [3.6%]). For grade 3–4 AEs, the incidence of febrile neutropenia (*p* = 0.03) and neutropenia (*p =* 0.04) in the SOX group was significantly higher than in the CIK-SOX group. No treatment-related deaths occurred.

**Table 2:** Adverse events.

Adverse event	N(%) Any grade		N(%) Grade 1-2		N(%) Grade 3-4		*P* value^*^
	CIK+SOX (n=31)	SOX (n=28)	CIK+SOX (n=31)	SOX (n=28)	CIK+SOX (n=31)	SOX (n=28)	
Leucopenia	15(48.4)	21(75.0)	15(48.4)	21(75.0)	0(0)	0(0)	–
Neutropenia	18(58.1)	18(64.3)	14(45.2)	8(28.6)	4(12.9)	10(35.7)	**0.04**
Febrile neutropenia	3(9.7)	9(32.1)	0(0)	0(0)	3(9.7)	9(32.1)	**0.03**
Fever	3(9.7)	2(7.1)	2(6.5)	2(7.1)	1(3.2)	0(0)	–
Anemia	16(51.6)	19(67.9)	15(48.4)	17(60.7)	1(3.2)	2(7.1)	0.50
Thrombocytopenia	13(41.9)	19(67.9)	10(32.3)	18(64.3)	3(9.7)	1(3.6)	0.36
Nausea	19(61.3)	22(78.6)	19(61.3)	22(78.6)	0(0)	0(0)	–
Vomiting	9(29.0)	12(42.9)	9(29.0)	12(42.9)	0(0)	0(0)	–
Decreased appetite	20(64.5)	22(78.6)	20(64.5)	22(78.6)	0(0)	0(0)	–
Diarrhea	0(0)	2(7.1)	0(0)	2(7.1)	0(0)	0(0)	–
Elevated ALT	10(32.3)	10(35.7)	10(32.3)	10(35.7)	0(0)	0(0)	–
Elevated AST	9(29.0)	14(50.0)	9(29.0)	14(50.0)	0(0)	0(0)	–
Elevated TBIL	10(32.3)	19(67.9)	9(29.0)	9(32.1)	1(3.2)	1(3.6)	0.94
Elevated DBIL	13(41.9)	12(42.9)	12(38.7)	11(39.3)	1(3.2)	1(3.6)	0.94
Peripheral neurotoxicity	2(6.5)	5(17.9)	2(6.5)	5(17.9)	0(0)	0(0)	–
Elevated CREA	3(9.7)	0(0)	3(9.7)	0(0)	0(0)	0(0)	–
Fatigue	1(3.2)	1(3.6)	1(3.2)	1(3.6)	0(0)	0(0)	–
Oral mucositis	0(0)	1(3.6)	0(0)	1(3.6)	0(0)	0(0)	–
Dysacusis	0(0)	1(3.6)	0(0)	1(3.6)	0(0)	0(0)	–
Alopecia	0(0)	1(3.6)	0(0)	1(3.6)	0(0)	0(0)	–

^*^ The difference of grade 3-4 adverse events between SOX group and CIK-SOX group was analyzed.

SOX, S-1+oxaliplatin; CIK-SOX, CIK cells in combination with SOX; ALT, alanine aminotransferase; AST, aspartate aminotransferase; TBIL total bilirubin; DBIL direct bilirubin; CREA, creatinine. Bold represents P-values with statistical differences.

### PFS and OS

**Figure 2:**
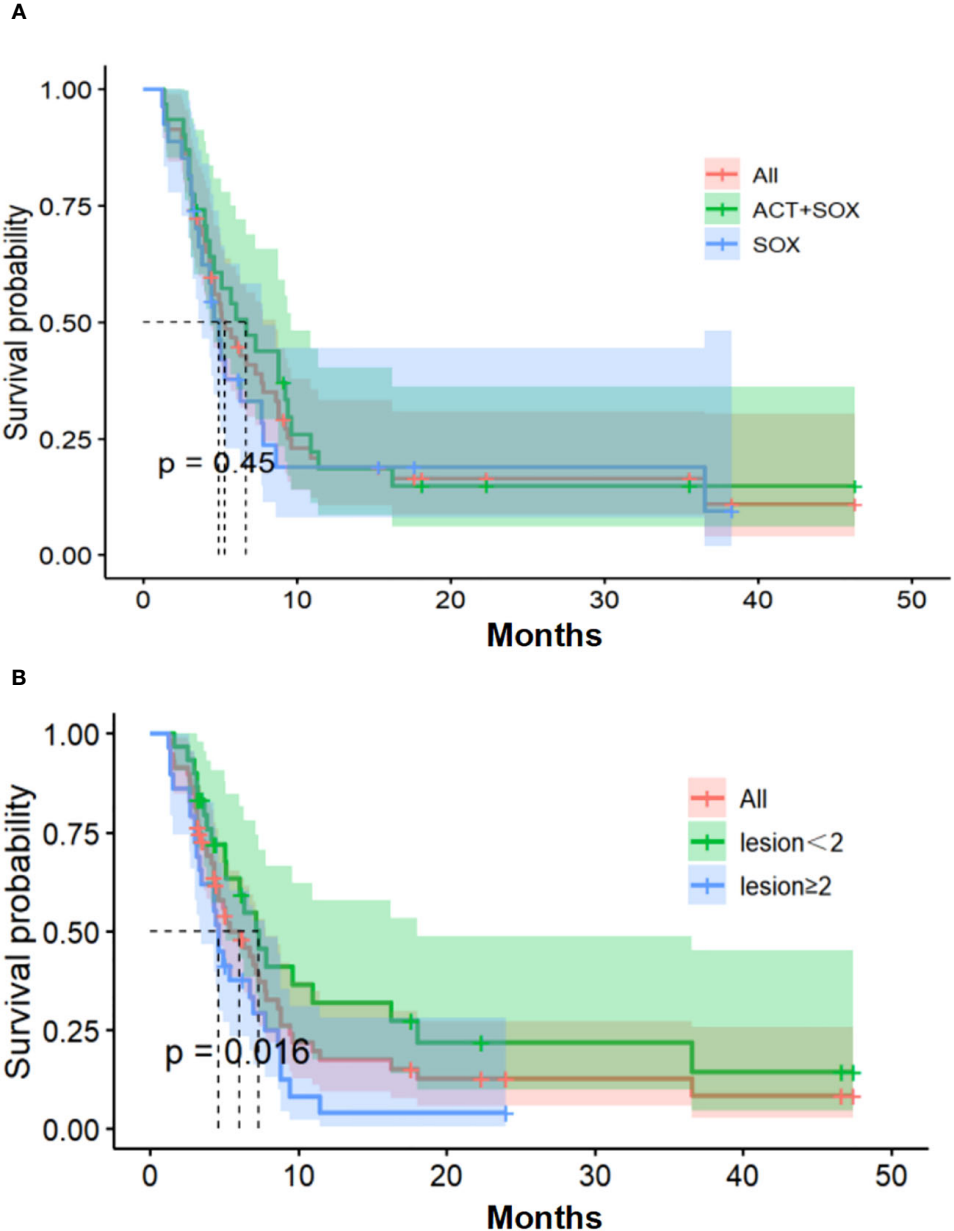
Progression-free survival. **(A)** Kaplan-Meier estimates of PFS. **(B)** The number of organs with tumor metastasis of PFS. PFS, progression-free survival.

As of December 6, 2019, 46 patients experienced endpoint events (PD) and 10 were censored (six patients received surgical treatment and four were lost to follow-up). Median PFS values were 6.9 months [95% confidence interval (CI), 4.3–8.8] in the CIK-SOX group and 4.9 months [95% CI, 3.6–7.7] in the SOX group (hazard ratio (HR) 0.80, *p*=0.45; **Figure 2**). The 6-month PFS rate was 51.6% in the CIK-SOX group and 32.1% in the SOX group. The respective 12-month PFS rates were 12.9% and 14.3%. The respective 18-month PFS rates were 9.7% and 7.1%. Subgroup analysis showed that tumor metastasis to more than two organs (HR 2.088, 95% CI 1.139–3.827, *p*=0.016) was an independent risk factor for PFS (**Figure 2**), while gender and age were not. Multivariate analysis showed that tumor metastasis to no fewer than two organs (HR 2.143, 95% CI 1.167–3.934, *p*=0.0139) and age (≥65 years old; HR 2.533, 95% CI 1.005–6.383, *p*=0.049) were risk factors for PFS.

**Figure 3:**
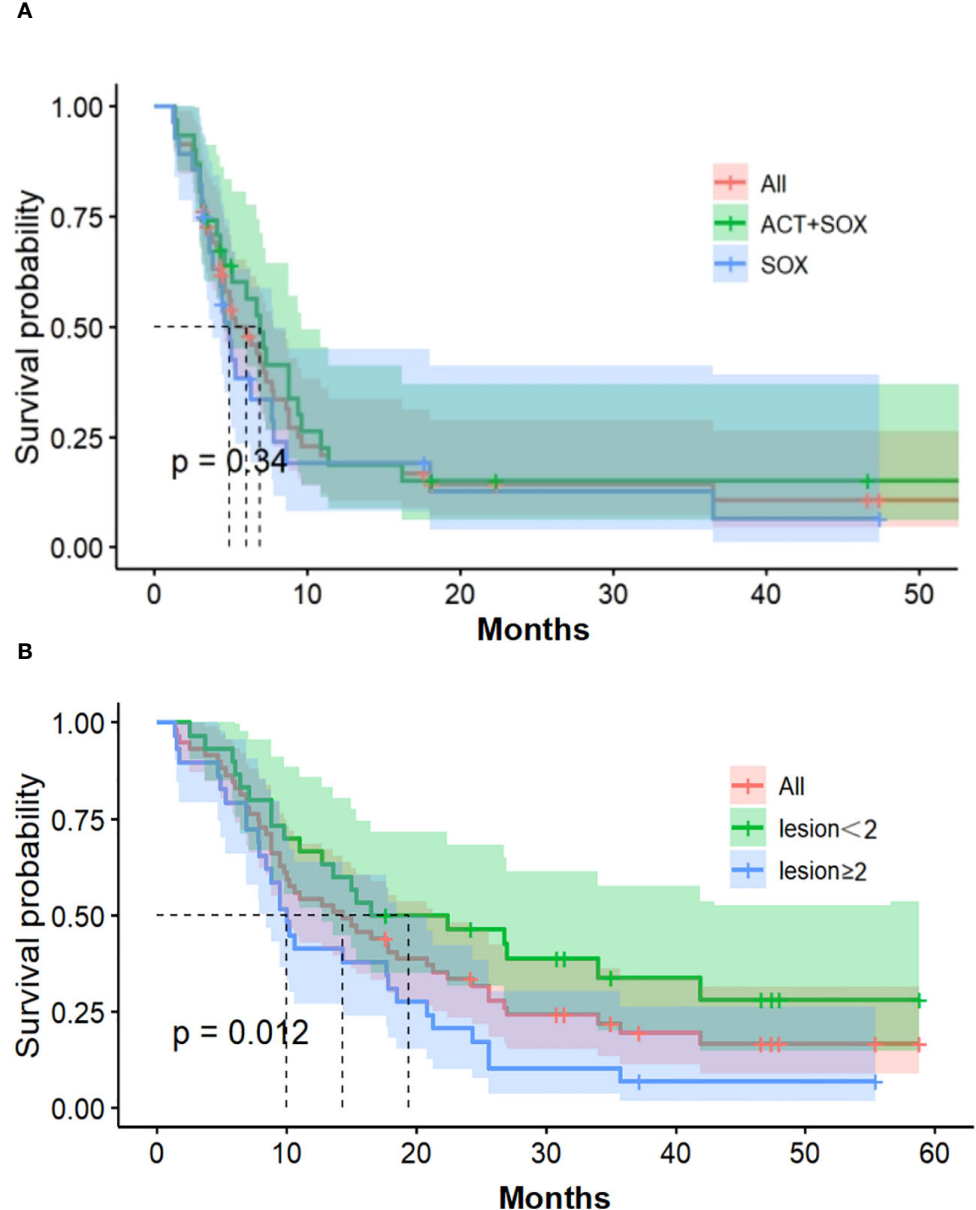
Overall survival. **(A)** Kaplan-Meier estimates of OS. **(B)** The number of organs with tumor metastasis of OS. OS, overall survival.

As of December 6, 2019, six patients in the CIK-SOX group and four patients in the SOX groups survived, and their median follow-up time was 47.3 and 41.2 months, respectively. Median OS for the CIK-SOX and SOX groups was 17.8 months (95% CI, 10.2–24.3) and 9.75 months (95% CI, 6.4–16.5; HR 0.76, *p*=0.34; **Figure 3**), respectively. The CIK-SOX group had a 12-month survival rate of 61.3%, and the SOX group had a 12-month survival rate of 42.9%. At 18 months, the OS rates were 48.4% and 28.6%, respectively (**Figure 3**). Subgroup analysis suggested that patients with tumors with metastasis to fewer than two organs (HR 2.091, 95% CI 1.161–3.765, *p*=0.012) were more likely to experience prolonged OS. Gender and age were not independent risk factors for OS. Multivariate analysis showed that tumor metastasis to no fewer than two organs (HR 2.056, 95% CI 1.129–3.742, *p*=0.018) was a risk factor for OS.

**Figure 4:**
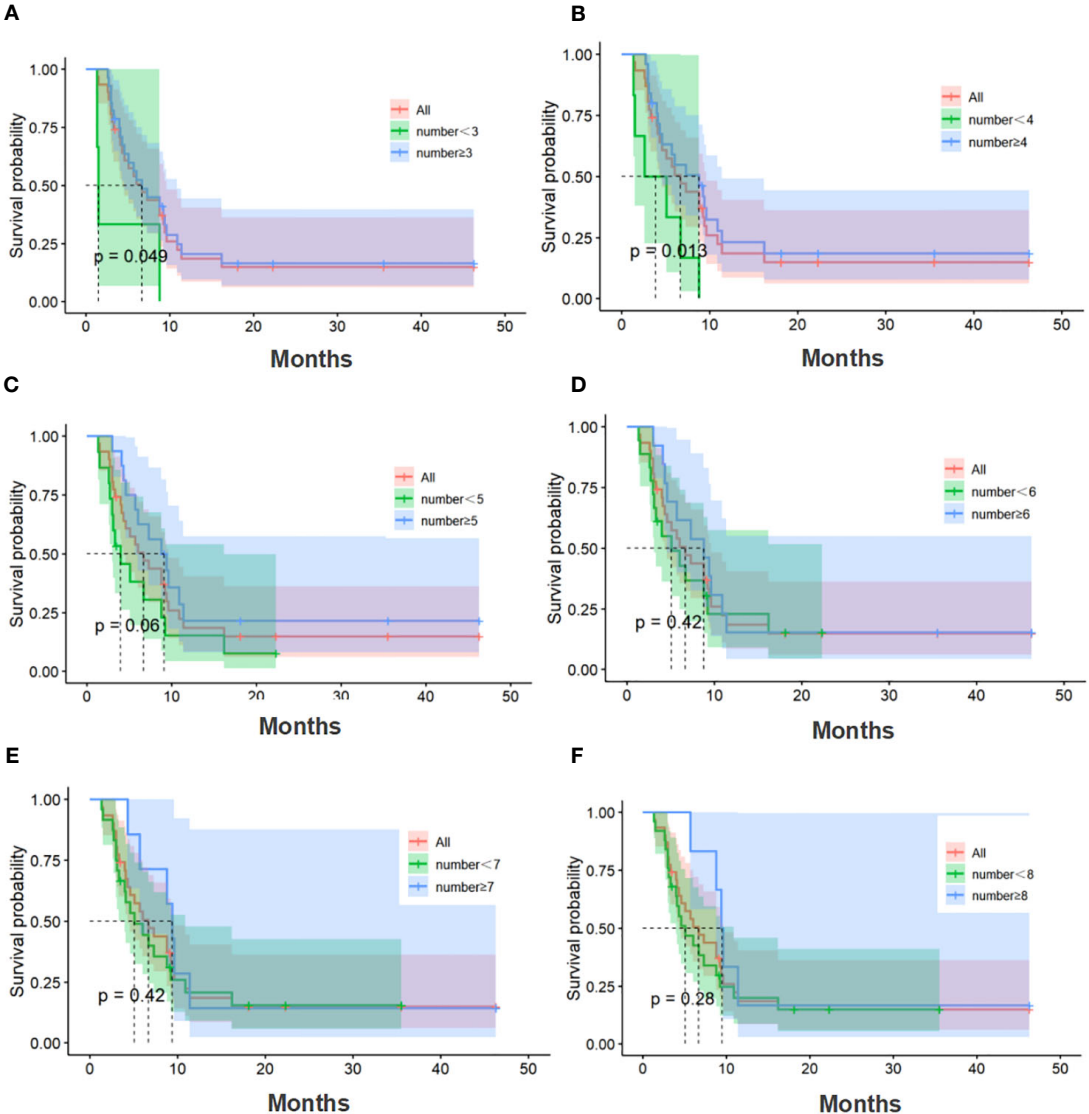
PFS of the number of cell therapy cycle. **(A)** Comparison of cell therapy cycle with no fewer than 3 cycles and fewer than 3 cycles. **(B)** Comparison of cell therapy cycle with no fewer than 4 cycles and fewer than 4 cycles. **(C)** Comparison of cell therapy cycle with no fewer than 5 cycles and fewer than 5 cycles. **(D)** Comparison of cell therapy cycle with no fewer than 6 cycles and fewer than 6 cycles. **(E)** Comparison of cell therapy cycle with no fewer than 7 cycles and fewer than 7 cycles. **(F)** Comparison of cell therapy cycle with no fewer than 8 cycles and fewer than 8 cycles. PFS, progression-free survival.

**Figure 5:**
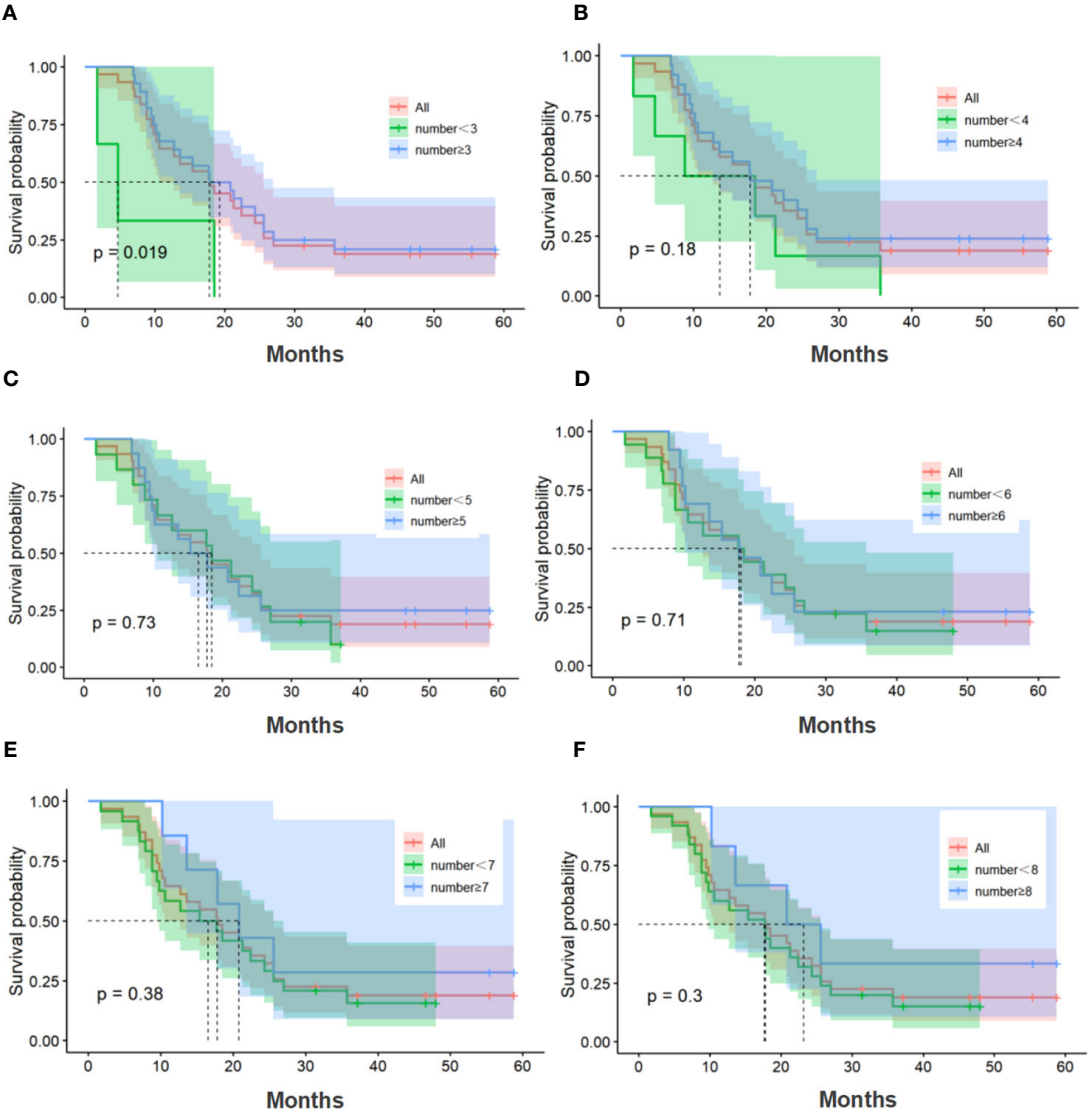
OS of the number of cell therapy cycle. **(A)** Comparison of cell therapy cycle with no fewer than 3 cycles and fewer than 3 cycles. **(B)** Comparison of cell therapy cycle with no fewer than 4 cycles and fewer than 4 cycles. **(C)** Comparison of cell therapy cycle with no fewer than 5 cycles and fewer than 5 cycles. **(D)** Comparison of cell therapy cycle with no fewer than 6 cycles and fewer than 6 cycles. **(E)** Comparison of cell therapy cycle with no fewer than 7 cycles and fewer than 7 cycles. **(F)** Comparison of cell therapy cycle with no fewer than 8 cycles and fewer than 8 cycles. OS, overall survival.

The median number of cell therapy cycle in the CIK-SOX group was 5. Twenty-eight patients with no fewer than 3 cell therapy cycles and twenty-five patients with no fewer than 4 cell therapy cycles. In terms of PFS (**Figure 4**), patients in the CIK-SOX group who received cell therapy ≥3 cycles (*p*=0.049) and ≥4 cycles (*p*=0.013) were better off than those receiving cell therapy <3 cycles and <4 cycles, respectively. In terms of OS (**Figure 5**), receiving cell therapy ≥3 cycles (p=0.019) was superior to receiving cell therapy <3 cycles in the CIK-SOX group.

### ORR and DCR

In the efficacy analysis, the investigators determined that 29 patients in the CIK-SOX group and 25 patients in the SOX group had measurable lesions. No patients in the CIK-SOX group achieved CR, while 16 patients achieved PR, and 11 patients achieved SD. The ORR was 55.2% (95% CI, 35.7–73.6%) and DCR was 93.1% (95% CI, 77.2–99.2%). In the SOX group, eight patients achieved PR and 14 patients achieved SD. The ORR and DCR were 32.0% (95% CI, 14.9–53.5%) and 88.0% (95% CI, 68.8–97.5%), respectively. The efficacy analysis results for all eligible patients are shown in **Table 3**.

**Table 3:** Best overall response (Patients with measurable lesions).

	CIK-SOX group N=29		SOX group N=25		P Value
	n (%)	95% CI	n (%)	95% CI	
Best overall response					
Complete Response (CR)	0	-	0	-	-
Partial Response (PR)	16(55.2)	-	8(32.0)	-	-
Stable Disease (SD)	11(38.0)	-	14(56.0)	-	-
Progressive Disease (PD)	2(6.9)	-	3(12.0)	-	-
Overall Response Rate (ORR: CR+PR)	16(55.2)	(35.7,73.6)	8(32.0)	(14.9, 53.5)	0.09
Disease Control Rate (DCR: CR+PR+SD)	27(93.1)	(77.2, 99.2)	22(88.0)	(68.8, 97.5)	0.88

N: The total number of subjects in the treatment group. It is the denominator for percentage (%) calculation. n: Number of subjects who are at the corresponding category. The exact 95% CI for the frequency distribution of each variable were computed using Clopper and Pearson method.

CR, complete remission; PR, partial remission; SD, stable disease; PD, progressive disease; ORR, objective response rate; DCR, disease control rate; CI, confidence interval.

## Discussion

With the development of ICIs, a number of large phase III clinical studies have established the role of ICI combined with chemotherapy in the treatment of locally advanced or metastatic GC patients, but the population-wide data showed limited benefits. It has been hypothesized that one of the reasons for the poor efficacy of anti-PD-1/anti-PD-L1 therapy is the limited T lymphocyte infiltration in the tumor mcroenvironment (22). Adoptively providing *in vitro* activated cell products, such as DCs, NK cells, or T cells, is a potential therapeutic option. A previous study has shown that the introduction of sufficient lymphocytes to recognize and decompose tumor cells is the basis of a successful ACT (23). Some studies have explored the efficacy and safety of ACT combined with different forms of chemotherapy regiments for GC patients. Liu et al. confirmed that DC-CIK combined with oxaliplatin-5-fluorouracil chemotherapy can improve immune cell function and prolong survival time in locally advanced GC patients (18). Qiao et al. found that DC-CIK combined with S-1 + cisplatin provided good PFS and OS in patients with advanced GC, and the adverse events were tolerable (16).

The present exploratory, random, open-label study investigated the use of autologous CIK cells combined with SOX versus SOX alone. Adding autologous CIK cells to SOX reduced the incidence of AEs. Previous studies have shown that activated CIK cells *in vivo* can secrete a variety of cytokines, such as IFN-γ, which can activate neutrophils (24–26). In addition, a large number of T lymphocytes increased the production of interleukin 6 (IL-6), a cytokine typically associated with inflammation. IL-6 promotes the release of neutrophils from the bone marrow and enhances their adhesion and aggregation (27, 28). Maybe due to the continuous release of rapidly growing numbers of neutrophils to the peripheral blood, the peripheral hemogram accelerate recovery. It can also be seen in our study that the incidence of febrile neutropenia and neutropenia in the CIK-SOX group was significantly lower than that in the SOX group. But more research is needed to validate this viewpoint. Moreover, autologous CIK cells combined with SOX showed a tendency to improve the median PFS and OS in patients with locally advanced or metastatic GC, although this result was not statistically significant. This may have been limited by the sample size. However, when SOX was combined with the autologous CIK cells, the absolute OS improvement time was about 8.05 months and safety was manageable. In addition, sustained benefits of autologous CIK cells in combination with SOX were observed through long-term follow-up, with higher PFS and OS rates observed at 12 and 18 months.

In previous studies, patients with a higher tumor load had a higher number of immunosuppressive cells, such as tumor-associated macrophages, regulatory T cells, or myeloid-derived suppressor cells, which promoted immune evasion while impeding immune monitoring (29). In addition, the expression of PD-L1 on tumor cell surface or peripheral blood serum may help to inhibit the function of tumor-specific T cells (30). Although transgenic T cells with a CAR or TCR can overcome immune tolerance for tumor antigens, this treatment is not effective against tumor cells that have lost the expression of specific epitopes targeted by CARs or TCRs. However, the interaction between tumor cells and immune cells during chronic inflammation may create more favorable conditions for the survival of tumor cells (31, 32). Therefore, fast proliferation, strong tumor killing activity, and a broad spectrum of tumor killing immune cells can effectively eliminate tumors. Moreover, studies have shown that chemotherapy-resistant cancer cells are very sensitive to the cytotoxic effects of ACT-lymphocytes (33, 34). Therefore, ACT-lymphocytes have the potential to eradicate residual tumor cells after chemotherapy. The autologous CIK cells was used in combination with chemotherapy in the present study, with the expectation that survival benefits could be further improved through possible synergies. This study is slightly different from the treatment regimen in previous studies (16–18), and we conducted a correlation analysis between different cell therapy cycles and survival time.

The present study results suggest that patients who received no fewer than three cycles of autologous CIK cells benefited the most from combination therapy. The timing of administration of the enhanced CIK cell preparation and favorable auto-lymphocyte characteristics may account for the improved OS and immune response. Treatment compliance in the study was satisfactory, which may be due to the low rate of serious AEs and well-tolerated characteristics, suggesting the feasibility of using autologous CIK cells in combination with chemotherapy. Subgroup analysis further suggested that the effect of autologous CIK cells may be more pronounced in patients with no fewer than two metastatic organs. This suggests that for patients with more extensive metastasis, improving the immune status of the body is more beneficial than chemotherapy alone, which needs to be confirmed by larger studies.

The present study had several limitations. This was an exploratory study with a relatively small sample size and safety serving as the primary endpoint. Moreover, the single-center study lacked sufficient universality, making it difficult to generalize its findings. Due to the early start year of the study, biomarkers, such as TMB and PD-L1, have not been stratified, and partial immunohistochemical results, including those for HER2, are missing. Different marker states may affect the final results. In addition, the level of lymphocytes represents the state of immune function in the body, and further analysis of the number of lymphocyte subsets in patients was not available in the present investigation. Multicenter randomized controlled trials in larger cohorts are needed to further validate the efficacy and safety of the combination regimen and to further screen the population for benefit analysis.

Future research should include determining the optimal number of cell therapy cycle and the interval between CIK cell treatments. In addition, population screening can be performed based on different markers, such as microsatellite status or PD-L1 expression, in order to explore the treatment efficacy in different types of patients receiving cell therapy.

## Conclusions

Autologous CIK cells in combination with SOX demonstrated a good safety profile as the first-line therapy in locally advanced or metastatic GC patients. Although the PFS and OS in the CIK-SOX group were not statistically significantly different compared to those in the SOX alone group, the treatment prolonged the PFS and OS duration, with the absolute improvement in OS duration of about 8.05 months. Continuous benefit of autologous CIK cells in combination with SOX was observed during long-term follow-up.

## Data availability statement

The original contributions presented in the study are included in the article/supplementary material. Further inquiries can be directed to the corresponding author.

## Ethics statement

The studies involving humans were approved by The Ethics Committee of the National Cancer Center/Cancer Hospital, the Chinese Academy of Medical Sciences and Peking Union Medical College. The studies were conducted in accordance with the local legislation and institutional requirements. The participants provided their written informed consent to participate in this study.

## Author contributions

XM: Conceptualization, Data curation, Writing – original draft, Formal Analysis, Methodology, Project administration. LP: Data curation, Formal Analysis, Writing – original draft. JW: Data curation, Formal Analysis, Writing – original draft. LG: Investigation, Writing – original draft. WZ: Investigation, Writing – original draft. XL: Methodology, Project administration, Writing – original draft. JL: Methodology, Project administration, Writing – original draft. LY: Project administration, Writing – review & editing.
